# Corrosion Resistance and Electrical Conductivity of Hybrid Coatings Obtained from Polysiloxane and Carbon Nanotubes by Electrophoretic Co-Deposition

**DOI:** 10.3390/ijms23052897

**Published:** 2022-03-07

**Authors:** Patryk Bezkosty, Elżbieta Długoń, Maciej Sowa, Jacek Nizioł, Piotr Jeleń, Jakub Marchewka, Marta Błażewicz, Maciej Sitarz

**Affiliations:** 1 Faculty of Materials Science and Ceramics, AGH University of Science and Technology, al. Adama Mickiewicza 30, 30-059 Kraków, Poland; dlugon@agh.edu.pl (E.D.); pjelen@agh.edu.pl (P.J.); jmar@agh.edu.pl (J.M.); mblazew@agh.edu.pl (M.B.); msitarz@agh.edu.pl (M.S.); 2 Faculty of Chemistry, Silesian University of Technology, ul. Marcina Strzody 9, 44-100 Gliwice, Poland; maciej.sowa@polsl.pl; 3 Faculty of Physics and Applied Computer Science, AGH University of Science and Technology, al. Adama Mickiewicza 30, 30-059 Kraków, Poland; niziol@agh.edu.pl

**Keywords:** polysiloxanes, carbon nanotubes, nanocomposites, coatings, electrophoretic co-deposition

## Abstract

Nanocomposites developed based on siloxanes modified with carbon nanoforms are materials with great application potential in the electronics industry, medicine and environmental protection. This follows from the fact that such nanocomposites can be endowed with biocompatibility characteristics, electric conductivity and a high mechanical durability. Moreover, their surface, depending on the type and the amount of carbon nanoparticles, may exhibit antifouling properties, as well as those that limit bacterial adhesion. The paper reports on the properties of polysiloxane (PS) and carbon nanotubes (CNT) nanocomposite coatings on metal surfaces produced by the electrophoretic deposition (EPD). A comparison with coatings made of pure PS or pure CNT on the same substrates using the same deposition method (EPD) is provided. The coatings were examined for morphology and elemental composition (SEM, EDS), structural characteristics (confocal Raman spectroscopy), electrical conductivity and were tested for corrosion (electrochemical impedance spectroscopy-EIS, potentiodynamic polarization-PDP). The results obtained in this study clearly evidenced that such hybrid coatings conduct electricity and protect the metal from corrosion. However, their corrosion resistance differs slightly from that of a pure polymeric coating.

## 1. Introduction

Carbon nanoforms are materials that have been widely described and studied, not only for their unique physical and chemical properties but also for their potential use as additives that modify the properties of metallic, ceramic and polymer matrices [1,2,3,4,5,6]. A polymer matrix combined with suitably prepared carbon nanotubes (CNT) form materials that typically have novel properties that can be highly customized. Nanoadditives also modify the surface properties of the final material, changing its topography and chemical state. In this way, degradation-resistant, conductive nanocomposites with better mechanical parameters than the starting material can be produced. Carefully selecting the way CNT are modified is essential to produce composites with properties that are impossible to obtain using conventional materials [7,8,9,10,11].

Usually, the key issue is the choice of the carbon-surface modification method of CNT. It must be appropriately adapted to the type of polymer, allowing the desired dispersion of the carbon additive to be achieved. [12,13,14]. In particular, the chemical compatibility of the CNT surface and the polymer matrix is vital to obtain materials characterized by electrical conductivity. If this condition is fulfilled, the fully conductive coatings can contain a very small fraction of CNT, already sufficient to exceed the percolation threshold [15,16]. Such coatings, including that made of other carbon nanoforms such as graphene or graphene oxide are among the prospective materials that can be used in many areas such as electronics, optoelectronics, environmental protection or medicine [17,18,19,20,21,22,23,24]. Moreover, it is possible to use them to manufacture multi-layered compositions with a dielectric of interesting capacitive properties on conductive or insulating substrates [25,26]. The modified coatings discussed in this paper demonstrate an additional asset because they can replace classic anti-corrosion coatings applied on metals. On the one hand, they are electrically conductive materials, while on the other they protect the underlying metal against corrosion. They present a great advantage, as corrosion products are not necessarily environmentally friendly. Test results from the available literature show that coatings also extend product life, limiting the occurrence of defects, which often promote the corrosion of the metal [27,28,29,30]. Nevertheless, obtaining coatings that simultaneously meet the requirements for electrical conductivity and improve corrosion resistance is a complex issue. Despite many literature reports presenting fully functional coatings, these materials are still not widely used in practice because of insufficient knowledge of related physics and chemistry. In this area of research, it seems particularly important to characterize and further analyze the phenomena accompanying the formation of such compositions. Especially, in terms of the formation of interfacial connections and the mechanism of interaction of the polymer (non-conductive phase) with the surface of the carbon phase (CNT) with high electrical conductivity.

The subject of many current studies are the coatings based on polysiloxanes modified with carbon nanoforms applied on various metallic substrates. Polysiloxanes (PS) are organic–inorganic polymers with different carbon atom substituents in the main chain. Therefore, there is a possibility of modifying their structure, as well as the physical and chemical properties. They are thermally and chemically resistant materials with low surface energy and surface hydrophobicity. They have enjoyed the unyielding scientific interest of many research groups since the 1960s and have already found a whole range of industrial applications, including construction, the automotive industry or medicine. Polysiloxanes are commonly used for the production of protective coatings with increased chemical and thermal resistance, including the production of the coatings applied to metals, additionally characterized by extremely low toxicity [31,32,33,34,35,36]. It is known that the functional properties of polysiloxanes coatings applied to metal surfaces (in addition to many factors concerning the amount and the type of carbon nanoforms introduced into the polysiloxanes) are influenced by the type and preparation of the metal substrate, as well as the procedure of manufacturing the polymer-carbon nanocomposite coating. One of the methods used to apply the coatings to metallic surfaces is the electrophoretic deposition. In this method, the charged particles (e.g. carbon nanotubes) dispersed in a suspension reach the surface of the electrode metal under the influence of an electric field. With the development of nanotechnology, it has been proved to be particularly useful, because it allows the relatively easy control of many process parameters. These factors are crucial for the final structure, microstructure and other functional properties of the resulting substrate/coating system. In our previous research [37,38], we developed a new method for the production of the coatings using electrophoretic co-deposition. It is based on the application of the suspension prepared in the form of polysiloxane sol with dispersed carbon nanotubes and their simultaneous deposition on the metal electrodes. This method allows for the production of material in which CNT formed an interconnected network system, tightly surrounded by the polymer matrix. The polymer component of the coating is responsible not only for the adhesion of the hybrid system to the metallic substrate, but also protects the entire system against corrosion. In such an electroconductive nanocomposite, the disadvantages resulting from poor adhesion of pure CNT coatings are eliminated.

The aim of the current study was to further investigate the nanocomposite coatings, produced in the EPD process from polysiloxane and CNT. The conductivity of the nanocomposite coating was determined and compared with that of a coating made of multi-walled carbon nanotubes alone. The corrosion resistance of the nanocomposite coating was compared to that of a PS coating. It is known that coatings made of CNT alone are materials of high electrical conductivity, and polysiloxane coatings are characterized by a high corrosion resistance. In our study, we show that the EPD co-deposition method creates particularly favorable conditions for manufacturing complex composite materials. The obtained properties of these materials indicate that this method allows, to a greater extent than in case of the other methods, for the transference of the properties of the components to the resulting material and to obtain the material with expected functional properties. They are derived from the properties of the phases incorporated in the nanocomposite coating.

## 2. Results

### 2.1. Characterization of Structure and Microstructure

In order to determine the morphology and the chemical composition, the obtained materials were tested by Scanning Electron Microscopy (SEM) with an Energy Dispersive Spectroscopy (EDS) system.

**Figure 1:**
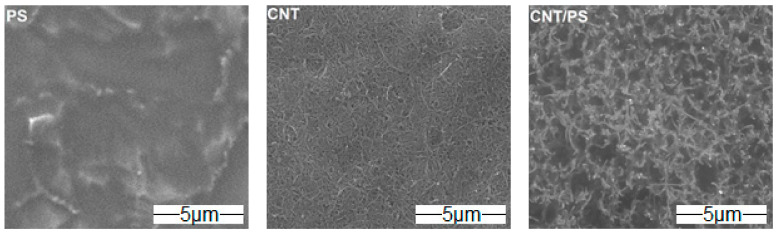
SEM images of PS, CNT and CNT/PS coatings on titanium surface.

The SEM images (Figure 1) show the coatings made of pure PS and CNT as well as the CNT/PS composite coating. CNT form the compact system of densely packed nanotubes interconnecting each other. The image of the nanocomposite coating is different. The surface of the coating shows CNT joining together, similar to the CNT layer, but between these nanocarbon agglomerates, polysiloxane filled areas are visible. It can be concluded that the CNT coating is characterized by specific nanotopography related to the presence of CNT whereas the topography of the nanocomposite coating is complex. For all the samples, the elemental composition was assessed by EDS (Table 1). Titanium originated from the applied surface. For the coating made of pure PS, the presence of silicone, oxygen and carbon was confirmed. The analysis of the CNT coating indicated the initial functionalization of CNT. Therefore, oxygen was identified alongside carbon. For the composite coating, the elements specifically required for both pure materials were demonstrated.

### 2.2. Raman Spectroscopy

**Figure 2:**
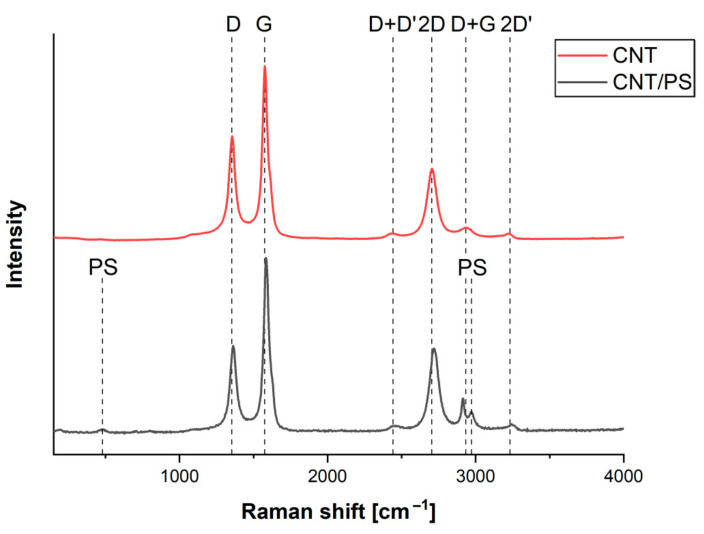
Normalized averaged spectra of pure CNT and co-deposited CNT/PS.

In order to determine the structure of the obtained layers, Raman spectroscopic studies were carried out. The averaged spectra are presented in Figure 2.

**Figure 3:**
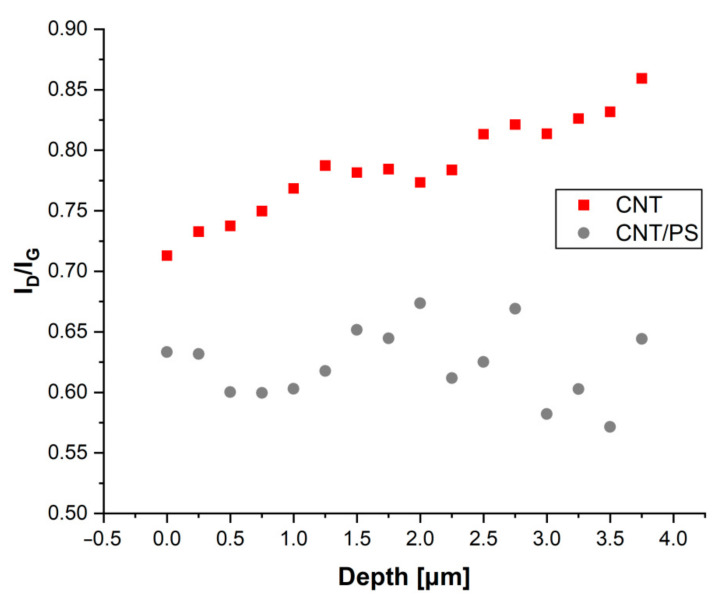
The I_D_/I_G_ ratio plotted against depth of measurement.

The presented spectra exhibit characteristic spectral features of the CNT and the CNT/PS composite as shown in our previous study [38]. The presented spectra exhibit typical bands for carbon nanotubes. As can be seen, the first order vibrations at approx. 1300–1600 cm^−1^ correspond to the carbon D (1355 cm^−1^) and G (1575 cm^−1^) bands. The first one, D band, represents the symmetric breathing of hexagonal carbon rings [39], while the second is attributed to the in-plane bond stretching of sp^2^ hybridized carbon pairs [40]. In addition, second order bands are also present. The existence of the 2D band, at 2702 cm^−1^, which corresponds to the breathing vibrations of six carbons pertaining to a hexagon in the hexagonal carbon lattice confirms the presence of CNT [41]. The lower spectrum (black one) exhibits additional feature-bands at 490, 2913 and 2971 cm^−1^ all of which can be attributed to the polysiloxane material [37]. The first originates from the Si-O vibration, while the other two originate from the C-H stretching vibration in Si-CH_3_ [37]. According to Ferrari and Robertson [42], in order to determine the structural changes of CNT material and the CNT/PS composite, the I_D_/I_G_ ratio must be calculated (intensity of D and G bands). To do this, spectra deconvolution of the D and G bands region was conducted for both samples. The obtained results were used to calculate the I_D_ and I_G_ parameters and are presented in Figure 3 as a depth function.

In both tested materials, there are some observed differences, which may be related to the value of the I_D_/I_G_ parameter. In the analyzed spectra, this parameter ranges from 0.55 to 0.85. In the case of the CNT/PS sample, its value, along with the distance away from the sample surface, fluctuates to a small extent and oscillates between 0.63 and 0.68. However, for the CNT sample, this parameter has the highest values in the spectra recorded at the shortest distance from the metal surface. This situation is typical for the nanocarbon coatings obtained using the electrophoretic deposition method. Carbon nanotubes are seen to form typically complex populations that demonstrate some variation in the degree of functionalization or length. Thus, it is likely that in the process of EPD, those are the most charged, and are most likely to be damaged firstly by reaching the metal surface. Such a phenomenon is not observed in the case of CNT electrophoretically deposited in the presence of the polysiloxane sol. This can probably be explained by the reaction occurring between the sol and oxygen groups on the surfaces of carbon nanotubes. Raman spectroscopy studies show that despite the fact that the number of nanotubes is significantly lower in the CNT coating compared to the coating made of pure CNT, in both cases, the carbon nanoforms forming the coatings are characterized by a high structural similarity, and therefore, a similar process of defecting the carbon phase and a similar process of the interactions taking place in the spatial systems was formed from CNT.

### 2.3. Electrical Conductivity

For both samples, it was found that their impedance was independent of frequency to approximately hundreds of kHz at all temperatures tested. It means that in the impedance measured, its real part was by far the dominant factor. Therefore, its value can be considered as the DC resistance. The measured resistance decreased with increasing temperature. Such a phenomenon is characteristic of systems with thermally activated charge transport, which occurs in many disordered systems. Models describing the resistance in such systems are often phenomenological and can be reduced to the Equation (1) [43]:

(1) $$R=R_{0}\exp{\left(\frac{T_{0}}{T}\right)}^{\alpha }$$

where $R_{0}$, $T_{0}$ and α are constants. The two latter can be related to activation energy and details on charge transfer mechanism between sites.

**Figure 4:**
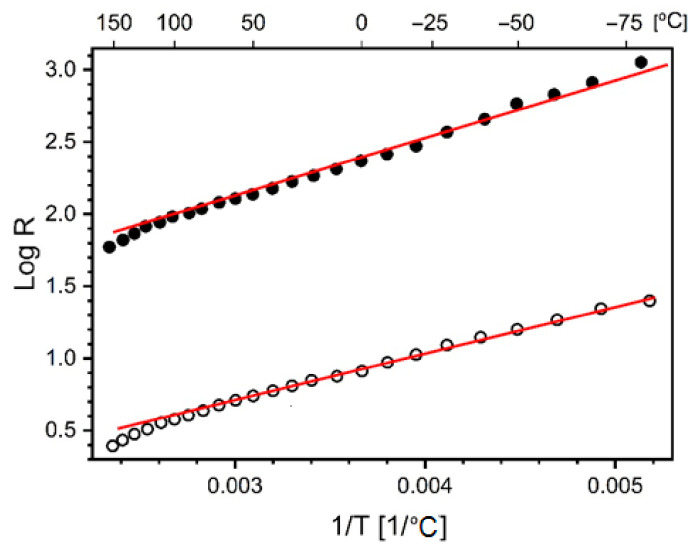
Logarithm of experimentally determined resistances as a function of the inverse of temperature, open circle–CNT coating, full circle CNT/PS coating. In these coordinates, the slope of the straight line segment of the graph corresponds to the α exponent value from Equation (1).

As shown in Figure 4, the resistances obtained for up to c.a. 130 °C are almost perfectly linear on a logarithmic scale as a function of the inverted temperature. In other words, the exponent α=1, is reflective of the classical Arrhenius type conductivity. In a bulk sample consisting of CNT, charge transport on a macroscopic scale is dominated by the transfer rate between individual nanotubes. It can be assumed that this rate depends on the temperature and energy barriers between the nanotubes, which occur mainly as a result of the spatial separation. The slope of the linear fits to the data for the CNT and CNT/PS coatings is virtually identical, despite the CNT/PS resistance being two orders of magnitude higher. Therefore, it can be assumed that the distribution of spatial barriers between CNT dispersed in the polysiloxane matrix is very similar to that observed in the layer consisting of CNT only.

### 2.4. Corrosion Resistance and Electrochemical Properties

**Figure 5:**
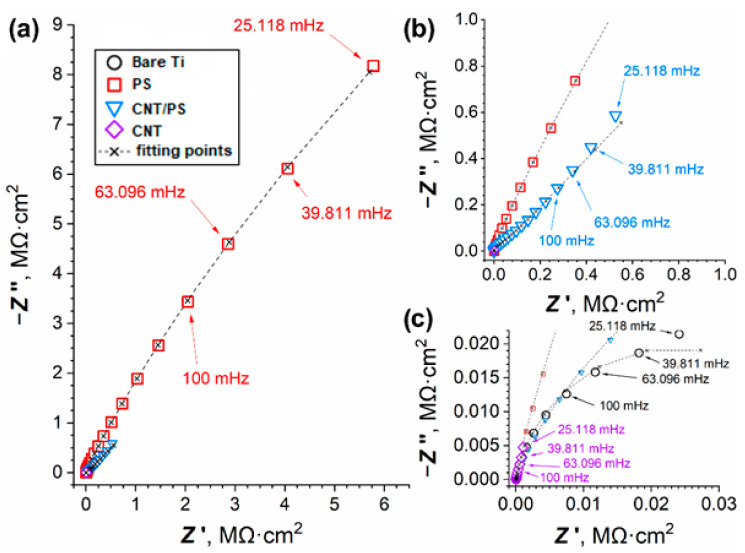
EIS spectra of the titanium samples coated with PS, CNT and co-deposited CNT/PS recorded in Ringer’s solution after 2 h of immersion presented as Nyquist plots at different scales (**a**–**c**) to show the observed capacitive loops.

**Figure 6:**
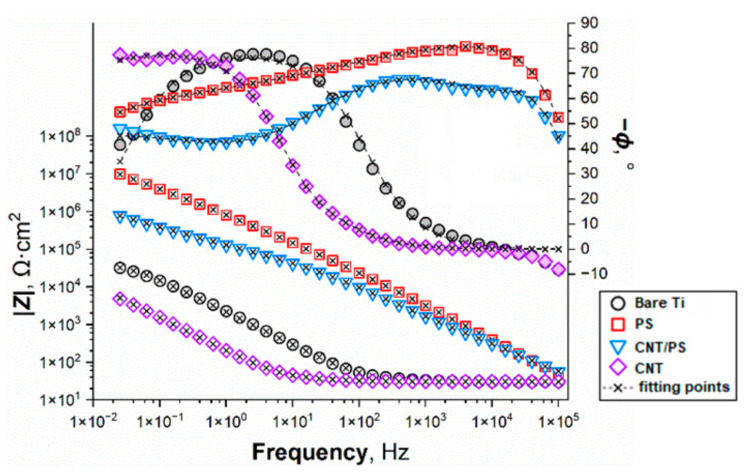
EIS spectra of titanium samples coated with PS, CNT and co-deposited CNT/PS recorded in Ringer’s solution after 2 h of immersion presented as magnitude (left axis) and phase (right axis) Bode plots. Empty symbols correspond to the impedance magnitude data, whereas the phase angle points are depicted as the shaded symbols.

To assess the corrosion resistance of the coated titanium specimens, electrochemical investigations were conducted. The first experiment that occurred was the EIS. The impedance spectra in the form of Nyquist (Figure 5) and Bode (Figure 6) plots clearly show that the most corrosion-resistant interface was that of the polysiloxane-coated titanium, as is evident from the high modulus and phase angles corresponding to this sample. For the bare titanium sample there is a single capacitive loop (Figure 5c and Figure 6) which corresponds to the RC pair in the *R_e_*(*Q_dl_R_ct_*) EEC. *R_e_* is the ohmic resistance of the electrolyte and electrode leads, *R_ct_* is the charge-transfer resistance of the corrosion reaction, whereas *Q_dl_* stands for the constant-phase element (CPE or *Q*), which is a general circuit element. The impedance of a CPE can be calculated from the Equation (2) [44]:

(2) $$Z_{\mathrm{CPE}}=1/Q{\left(j\omega \right)}^{n}$$

*Q* and *n* are the characteristic CPE parameters, *ω* is the angular frequency and *j* is the imaginary unity. When *n* attains values close to 1, the meaning of *Q* is somewhat reminiscent of the capacitance *C* (exactly the same at *n* = 1). CPEs are notoriously encountered in the corrosion studies because of their adaptivity to the fitting procedure, as they have an additional degree of freedom when compared with the ideal capacitance. The departure of the value of *n* from unity is ascribed to the non-uniformity of the electrochemical interface (in terms of the concentration of the electroactive species, roughness, chemical composition of the surface), which is commonplace for the corroding metal surfaces. Hence, *Q_dl_* represent the capacitive behavior of the electrical double layer of the studied interface. It is typical to adopt an *R*(*Q_c_*[*R_po_*(*Q_dl_R_ct_*)]) EEC for the defective coating in the corrosion medium. *Q_c_* models the capacitive properties of the coating and *R_po_* is the resistance of the electrolyte in its pores and defects. The additional circuit elements are used to fit the data to the additional capacitive loops arising from the coating defects. During the fitting procedure, it was found that the EEC corresponds to the experimental data of the PS and CNT-coated samples well (Figure 5 and Figure 6). However, in the case of the co-deposited polysiloxane and CNT, it was necessary to insert a Warburg element to the circuit to obtain a satisfactory fit with reasonable values. Therefore, for the CNT/PS samples an *R*(*Q_c_*[*R_po_*(*Q_dl_*[*R_ct_W*])]) was used. A similar circuit was used in our previous work in the case of the CNT-coated stainless steel surface [38]. The impedance of the Warburg element can be obtained from the Equation (3) [44]:

(3) $$Z_{W}=\sigma /\sqrt{\omega }-j\sigma /\sqrt{\omega }$$

where *σ* is the Warburg coefficient. The results of the fitting procedure can be found in Table 2. The values of the effective capacitance of the coating (*C_c_*) and the electrical double layer (*C_dl_*) were estimated with the use of the following Equation (4) [44,45]:

(4) $$C_{i}=\sqrt[{n}]{QR}/R$$

where *Q* and *R* are connected in parallel. *R_tot_* is the measure of the total resistance of the electrochemical interface when undergoing corrosion. The higher its value, the better the overall protective properties of the given sample series.

It can be noted from the impedance spectra of the polysiloxane-coated specimens (Figure 5a and Figure 6) that the negative phase angles are relatively high (between 50 and 85°), which is attributed to the good insulating properties of the deposited coating. *R_tot_* corresponding to this sample (Table 2) is the highest among the studied interfaces (60.8 ± 23.9 MΩ·cm^2^). It shows that the degree to which the defects in the coating are compromising its protective properties is not significant. On the other hand, the CNT-coated titanium surfaces were found to be rather ineffective at protecting the substrate from the ingress of the Ringer’s solution. The presence of the CNT can cause the electrocatalytic effect in the oxygen reduction reaction (ORR) [46,47] which is a depolarization reaction for the oxidation of the substrate. It is also evident that the surface area of the CNT contributes to the high capacitance of the coating (Table 2). At the same time, the *R_po_* is evidences the low ability of the coating to shield titanium from the electrolyte. Moreover, the value of *R_tot_* obtained for the CNT-coated samples is only slightly higher than that of the bare titanium. Therefore, the CNT coating is not effective at boosting the corrosion resistance of titanium. The spectrum of co-deposited CNT/PS coating (Figure 6) shows that its impedance response is a mixture of the pure CNT and PS coatings. The presence of the CNT in the coating demonstrably increases the abundance of defects in the polysiloxane structure, thereby reducing its insulating properties. However, the ingress of the electrolyte and the dissolved oxygen towards the titanium surface is much harder, which is observed as high values of *R_tot_* and *σ* (Table 2). Sai Jyotheender and Srivastava [48] investigated the effect of the concentration of the CNT in the solution for the electrodeposition of Zn coatings on the corrosion resistance of the mild steel Zn/CNT-coated specimens. It was shown that in the intermediate CNT concentration range, the corrosion resistance of the resulting interfaces was higher than the pure Zn coatings, whereas the highly concentrated solutions led to the deposition of the surface layers with poor protective capabilities. It was postulated that the reason for this was that the microstructure (Zn crystals orientation) of the deposited coatings was dependent on the CNT concentration. However, Zn coatings have a sacrificial character because they are thermodynamically preferable to undergo corrosion than most substrates (e.g., steel, titanium). Therefore, improving the electrical contact between the Zn crystals and the substrate are conducive to better protection [49]. Nevertheless, there were some successful applications of the composite coatings with the use of CNT with polyaniline [50] and with the same polymer combined with CNT and some ionic liquids used as an additive to the epoxy coating [51]. The addition of the CNT brought about the more protective character to the coatings. However, in the case of this type of coatings, the deposition occurred through the electropolymerization route.

**Figure 7:**
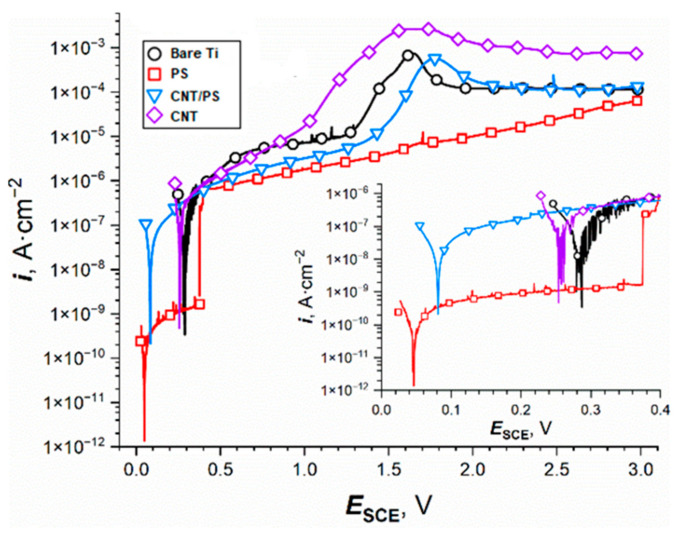
The potentiodynamic polarization curves obtained for the titanium specimens with different coatings in Ringer’s solution. The inset corresponds to the same data but at a finer scale.

After the EIS measurements, PDP curves of the studied samples were recorded (mostly) in the anodic region (Figure 7) to investigate their ability to protect the substrate in the oxidizing environment.

Some data were collected from the curves in the form of corrosion potential (*E_corr_*), coating breakdown potential (*E_bd_*), and current density values at the potential of 1 or 3 V vs. SCE (Table 3) to make the comparison between the samples more convenient. Because titanium is prone to passivation in chloride environments [52] (e.g., Ringer’s solution), upon polarization it was impossible to assess the corrosion current density of the samples. The application of the polysiloxane coating on titanium led to a shift in its corrosion potential from about 330 mV vs. SCE to values close to 0 mV vs. SCE. It might be rationalized by the inhibiting effect of the ORR rate caused by the presence of the insulating coating, because the bare titanium corrosion rate is typically limited by the anodic reaction (passivation of the substrate). A similar effect, but to a lesser extent, was encountered for the CNT/PS coatings (Figure 7, Table 3).

The corrosion potential of the CNT-coated specimens was only slightly lower than that of the bare titanium surface, probably because the passivation of the substrate was limited due to the presence of the CNT on top of the metal surface. Interestingly, the superior corrosion properties of the polysiloxane-coated titanium samples were maintained only until a potential of about 380 mV vs. SCE was reached, because of the vast current density increase that signifies the failure (breakdown) of the coating and the uncovering of the bare substrate underneath. The remainder of the curve is still below the curves corresponding to the other samples, however, not by a high margin (Figure 7, Table 3). In the case of the CNT-coated titanium, the PDP curve follows that of the bare titanium sample until the potential of about 1 V vs. SCE, which is the onset potential of oxygen evolution (Figure 7). At this point, the electrocatalytic effect of the CNT is exhibited once again. As it was encountered for the EIS results, the PDP curve of the co-deposited CNT/PS coating displays a behavior that is in between the CNT and polysiloxane samples. There were no visible marks of the coating breakdown during the measurement (Figure 7); however, the values of the current density at the beginning of the scan were at a level similar to that after the breakdown (Table 3), signifying that the failure of the coating occurred without anodic polarization. It is also worth noting that the error associated with the EIS results (Table 3) in the case of the polysiloxane and CNT/PS samples was relatively high, meaning that during the experiment, the coatings could undergo changes leading to their eventual failure (breakdown). Nevertheless, even after the coating failure, the corrosion resistance of the studied interface was still found to be better than that of the bare titanium. Moreover, there is a need for the further optimization of the amount of CNT in the coatings to push the corrosion resistance of the system beyond the pure PS coatings. For example, it was found by Zhang et al. [53] that there is an optimal amount of CNT in the hydroxyapatite/CNT composite that improves the corrosion resistance of the system the most.

## 3. Materials and Methods

### 3.1. Preparation of Polysiloxane Sol

Methyltriethoxysilane (MTES, ≥98%, Sigma-Aldrich, St. Louis, MO, USA) and dimethyldiethoxysilane (DMDES, ≥97%, Sigma-Aldrich, St. Louis, MO, USA) were used as precursors for sol-gel synthesis. The carbon content of the final product can be effectively controlled by the ratio of these reactants containing two different siloxane units, referred to as T units (with a ratio of carbon, oxygen and silicon atoms = 3:1:1) and D units (with a ratio of carbon, oxygen and silicon atoms = 2:2:1) present in MTES and DMDES, respectively. Both precursors were mixed in a 2:1 molar ratio with the assumption to obtain a ladder-like structure that we described in our previous papers [39,40,54]. Ethanol (99.8%, Avantor Performance Materials, Gliwice, Poland) was used as the solvent and the whole mixture was hydrolyzed with diluted (pH = 4.5) 1 M hydrochloric acid (Avantor Performance Materials). A clear colorless sol solution (with T:D = 2:1 ratio) was received after mixing for 6 h.

### 3.2. Preparation of CNT Solution

Coatings of carbon nanotubes (MWCNT–multi-walled carbon nanotubes, NanoAmor, Houston, TX, USA) ranging from 5–15 nm in diameter and 10–20 μm in length were deposited on titanium substrates. They were functionalized in a 3:1 mixture of concentrated sulfuric acid (Avantor Performance Materials) and concentrated nitric acid (Avantor Performance Materials) to remove metal particles, used as a catalyst during the preparation of nanotubes. Dissociation of the carboxyl groups formed in this way provide a negative charge that is necessary for electrophoretic deposition. A 4%(*w*/*w*) suspension of CNT was prepared in a mixture of isopropanol (≥99.5%, Avantor Performance Materials), acetone (≥99.5%, Avantor Performance Materials) and distilled water in a volume ratio of 3:1:1, respectively. The solution was sonicated for 10 min using an ultrasonic cleaner (Sonic-0.5, Polsonic, Warszawa, Poland) and directly used for electrophoretic deposition.

### 3.3. Preparation of Titanium Plates

Titanium plates (Gr2, 3.7035 per ASTM B 265 standard) with dimensions of 10 × 15 mm were used as the substrate for electrophoretic deposition. The procedure of substrate preparation began with mechanical cleaning, which consisted of grinding the plates with PS11 sandpaper of P600A gradation, followed by chemical cleaning in two consecutive steps:

1. immersion of the plates in acetone (≥99.5%, Avantor Performance Materials) and sonication for 15 min using an ultrasonic bath (Polsonic, model: Sonic-0.5),
2. immersion in ethanol (≥96%, Avantor Performance Materials) and sonication at the same conditions.

Finally, the plates were rinsed abundantly with distilled water and dried.

### 3.4. Preparation of Coatings

Three types of the coatings were prepared: CNT (pure), PS (pure) and CNT/PS (composite). All samples were manufactured on the titanium surface by electrophoretic deposition, and the third was prepared using a 1:1 (*v*/*v*) mixture of both multi-walled carbon nanotubes and polysiloxane solutions, known as co-deposition. The electrophoretic deposition procedure was carried out under the following conditions: one cycle, time: 30 s, voltage: 30 V, distance between electrodes: 10 mm. In order to deagglomerate the particles and improve their dispersion throughout the solution volume (which significantly affects the quality of the final products), immediately before the EPD process, both solutions were sonicated for 15 s using an ultrasonic homogenizer (Vibra-Cell VCX 500, Sonics & Materials, Newtown, CT, USA).

### 3.5. Scanning Electron Microscopy (SEM)

The microstructure and the chemical composition of the prepared materials were determined using Phenom XL (ThermoFisher Scientific, Waltham, MA, USA) equipped with the Electron Dispersive Spectroscopy (EDS) system. Before the examination, all samples were sputter coated with a thin gold layer (3 nm) using EM ACE200 vacuum coater (Leica, Wetzlar, Germany). Back-scattered Electrons Detector and the acceleration voltage of 10 kV (for SEM images) or 15 kV (for EDS analysis) were applied. The results of the EDS analysis were given as the average from four spots.

### 3.6. Raman Spectroscopy

Raman measurements were performed using WITec Alpha 300M+ spectrometer (WITec Wissenschaftliche Instrumente und Technologie GmbH, Ulm, Germany) equipped with a 488 diode laser and 600 grooves grating. The Z line scanning was performed to evaluate changes to the structure of the carbon nanotubes/polysiloxane composites. For this purpose, a set of parameters were chosen: a step of 0.25 um (to a total of 3.75 um depth) along with a 20 s integration time and 10 accumulations in each step. In order to evaluate the I_D_/I_G_ ratio spectral deconvolution was carried out using WITec ProjectFive Plus software. For ease of presentation, the obtained spectra were then averaged.

### 3.7. Electrical Conductivity

Preliminary tests showed a relatively high electrical conductivity of the prepared coatings. In such a case, a four-point technique is recommended to measure this parameter. Typically, four contact electrodes are applied on the top of the test sample. However, as the test coatings were relatively thin and were already deposited on a metallic substrate, measurements in in such geometry could lead to incorrect results. Because of this constraint, measurements were made using an alternating electric field at multiples frequencies. This technique, known as dielectric spectroscopy, can be used to determine DC conductivity if the resulting current does not depend on the frequency of the applied voltage. This is usually true at low frequencies. Furthermore, imperfect mechanical contact is not as critical as in the DC experiment.

The samples deposited on titanium plates were covered with a top, mechanically pressed, metallic electrode, which was smaller than the sample, to minimize stray field effects at the edges. To calculate specific electrical resistivity, it is necessary to know the precise value of the sample thickness. Due to the rough topography of the tested samples, it was not possible to determine this parameter unambiguously. However, the difference in the mechanism of electric charge transport is not only expressed by absolute values of resistivity. Even more instructive is the thermal evolution of resistance, which can be used as a criterion to indicate differences between samples.

The experimental setup consisted of an MFIA 5 MHz impedance analyzer (Zurich Instruments, Zurich, Switzerland) and a custom-made cryostat. Initially, the sample was cooled down to −80 °C, then equilibrated at temperatures subsequently raised by 10 °C up to 150 °C. The upper temperature was limited by the thermal stability of the sample, established as some 200 °C. At each temperature, the absolute impedance and loss tangent were measured. Measurements were carried out at a frequency range from 1 Hz to 1 MHz (in 10 steps per decade), applying a 300 mV_RMS_ test signal.

### 3.8. Corrosion Testing

Electrochemical methods were used to investigate the corrosion resistance of coated titanium samples in Ringer’s solution (8.6 g·dm^−3^ NaCl, 0.3 g·dm^−3^ KCl and 0.48 g·dm^−3^ CaCl_2_·6H_2_O; Fresenius Kabi, Kutno, Poland) at 37 °C. The experimental setup used for the measurements was described in our previous work [37]. The potentials were measured with respect to the saturated calomel electrode (SCE). The experimental protocol was prepared using dedicated VersaStudio (ver. 2.60.6) software and run by PARSTAT 4000 potentiostat-galvanostat (AMETEK Scientific Instruments, Princeton Applied Research, Berwyn, PA, USA). The following measurements were carried out in the procedure:

- Open-circuit potential (*E*_OC_) stabilization for a period of 2 h;
- Electrochemical impedance spectroscopy (EIS) at *E*_OC_ in the frequency range from 100 kHz to 25.118 mHz and the amplitude of 15 mV, recorded at five points per decade;
- Potentiodynamic polarization (PDP) from the potential of −50 mV vs. *E*_OC_ to 3000 mV vs. *E*_SCE_ at the scan rate of 0.167 mV·s^−1^.

Electrical equivalent circuits (EECs) served as a model of the studied interfaces for the sake of modeling the contributions of certain processes to the total measured impedance. A complex non-linear least square (CNLS) method was used for the fitting of the measurement EIS data to the circuits with the use of ZSimpWin (ver. 3.60, AMETEK Scientific Instruments) software. The measurements were completed in triplicate for each investigated sample and for the un-coated titanium specimens.

## 4. Conclusions

The coatings on metals obtained in the EPD co-deposition are of materials with valuable functional properties. They are characterized by both increased corrosion resistance and electrical conductivity. The CNT/PS coatings improve the anti-corrosion properties of titanium slightly less than when compared to pure polysiloxane. The results of Raman spectroscopy indicate that CNT functionalization takes place during the preparation process, whereas it has little effect on the electronic properties of carbon nano-addition. Thus, the nature of the conductivity of the CNT/PS coatings indicates that although its electrical resistance is much higher than that of the CNT coatings, this parameter can be changed depending on the amount of carbon nano-additive introduced into the polysiloxane. To sum up, co-deposition in the electrophoresis process can help to obtain coatings with the desired functionality, and this method can control the structure of the coatings in terms of the distribution of the particles in the polymer matrix.

## Figures and Tables

**Table 1:** EDS analysis results for the obtained coatings.

Element	Sample					
	PS		CNT		CNT/PS	
	[at%]	[wt%]	[at%]	[wt%]	[at%]	[wt%]
Si	15.60 ± 1.57	22.55 ± 1.77	-	-	15.18 ± 1.09	22.10 ± 1.60
O	56.34 ± 2.68	45.26 ± 2.42	8.97 ± 2.18	8.24 ± 2.21	45.29 ± 1.42	39.93 ± 2.52
C	18.99 ± 2.08	11.44 ± 1.64	76.55 ± 3.94	52.74 ± 4.40	31.98 ± 3.23	19.82 ± 2.87
Ti	8.64 ± 1.22	20.75 ± 1.32	14.48 ± 4.04	39.03 ± 5.29	7.57 ± 1.70	18.16 ± 3.75

**Table 2:** EIS fitting parameters used in the analysis of the impedance spectra of the titanium samples.

	Bare Ti	PS	CNT/PS	CNT
*Q*_c_, *s^n^* [MΩ^−1^·cm^−2^]	-	0.0514 ± 0.0199	0.0292 ± 0.0131	847 ± 71
*n*_c_	-	1.00 ± 0.01	1.00 ± 0.00	0.86 ± 0.01
*C*_c_ [μF·cm^−2^]	-	0.0517 ± 0.0204	0.0290 ± 0.0129	1800 ± 282
*R*_po_ [kΩ·cm^2^]	-	1.51 ± 0.18	0.572 ± 0.215	0.0458 ± 0.0078
*Q*_dl_, *s^n^* [MΩ^−1^·cm^−2^]	86.9 ± 3.6	0.360 ± 0.082	0.495 ± 0.284	183 ± 8
*n*_dl_	0.88 ± 0.00	0.65 ± 0.01	0.75 ± 0.03	1.00 ± 0.00
*C*_dl_ [μF·cm^−2^]	107 ± 2	1.82 ± 0.39	0.513 ± 0.293	183 ± 8
*R*_ct_ [MΩ·cm^2^]	0.0506 ± 0.0054	60.8 ± 23.9	0.151 ± 0.112	0.118 ± 0.037
*σ* [MΩ·cm^2^·s^−0.5^]	-	-	0.404 ± 0.234	-
*R*_tot_ [MΩ·cm^2^]	0.0506 ± 0.0054	60.8 ± 23.9	2.70 * ± 1.59	0.118 ± 0.037
*χ* ^2^	<3.28 × 10^−3^	<2.55 × 10^−4^	<7.30 × 10^−4^	<4.71 × 10^−4^

* For the calculation of *R*_tot_ of the CNT/PS sample the contribution from the Warburg impedance was assessed as the *σ*/(*ω*)^0.5^ with *ω* = 0.025118 (the lowest frequency in the experiment).

**Table 3:** Potentiodynamic polarization results in the form of corrosion parameters.

	Bare Ti	PS	CNT/PS	CNT
*E*_cor_ [mV] vs. SCE	329.2 ± 48.9	−9.2 ± 59.0	95.8 ± 21.2	240.7 ± 2.8
*E*_bd_ [mV] vs. SCE	-	383.4 ± 9.4	-	-
*i* @ 1 V vs. SCE [μA·cm^−2^]	7.42 ± 0.23	0.00154 ± 0.00010 *	2.86 ± 0.38	17.5 ± 0.2
*i* @ 3 V vs. SCE [μA·cm^−2^]	148 ± 28	75.0 ± 11.5	115 ± 23	708 ± 44

* Current density just before the coating breakdown.
